# Annual Meeting of the American Medical Association in St. Louis

**Published:** 1873-06-01

**Authors:** 


### MEDICAL EXAMINER.

*A Semi-Monthly Journal of Medical Sciences.*

EDITED BY

N. S. DAVIS, M. D., AND F. H. DAVIS, M. D.

Chicago, June 1st, 1873.

### EDITORIAL.

### ANNUAL MEETING OF THE AMERICAN MEDICAL ASSOCIATION IN ST. LOUIS, MAY, 1873.

The Association commenced its twenty-fourth annual session, in the Masonic Hall, St. Louis, at 11 o'clock A.M., May 6th, 1873. The retiring President, Dr. D. W. Yandell, of Louisville, Kentucky, introduced the Rev. Dr. Nichols, who opened the exercises with an appropriate prayer. Dr. John S. Moore, in behalf of the profession of St. Louis, cordially welcomed the members of the Association to the hospitalities of the city, and tendered free access to whatever could be found of medical interest in her schools, hospitals, and charitable institutions.

Dr. J. B. Johnson, Chairman of the Committee of Arrangements, after a few appropriate words of welcome, presented the report of the Committee in regard to the names of members registered, the invitations to various places of interest and entertainment, and the hours for holding the daily sessions. The recommendations of the Committee were adopted.

Dr. D. W. Yandell then introduced the President of the Association for 1873, Dr. Thomas M. Logan, of Sacramento, California. Dr. Logan, on taking the chair, presented his inaugural address, in which he presented and discussed several topics of importance, and was listened to with marked attention. It was much too long, however, occupying more than one hour and a half in the reading.

A motion was made, and adopted, tendering the thanks of the Association, and asking a copy for publication.

The President then appointed the Committee on Ethics, as follows: Drs. N. S. Davis, R. L. Howard, H. F. Askew, D. W. Yandell, and J. M. Toner, to which was referred such applications for membership as the Committee of Arrangements did not feel authorized to decide. The list of Special Committees was then called, and such reports and papers as were announced in readiness were referred to the appropriate Sections.

The morning session was then closed.

At 3 o'clock P. M., the Section on Surgery, and the one on Practical Medicine and Obstetrics, met and proceeded to business. Both were well attended, and were occupied in the consideration of topics of interest until 6 P.M. Owing to some imperfection in the arrangements for rooms, the other Sections accomplished but little. In the evening, the members, with their ladies and many citizens, enjoyed a very pleasant musical soiree in the hall of the Mercantile Library.

#### SECOND DAY'S PROCEEDINGS.

The Association was called to order at 9 o'clock, A. M., Dr. Logan in the chair.

The President recommended that each State appoint a delegate to represent them in the Committee on Nominations. The roll was called, and the following gentlemen named by their delegations respectively:

Arkansas, A. L. Brysacker; Alabama, G. Moses; Connecticut, W. A. Wainwright; District of Columbia, F. Howard; Georgia, J. P. Logan; Illinois, H. A. Johnson; Indiana, W. H. Meyers; Iowa, A. M. Carpenter; Kansas, D. W. Stormont; Kentucky, R. H. Gale; Maine, A. Gardlow; Maryland, S. P. Smith; Massachusetts, L. F. Warner; Delaware, H. F. Askew; Michigan, W. Brodie; Minnesota, A. B. Stewart; Missouri, J. B. Johnson; Mississippi, J. W. M. Shattuck; New Hampshire, E. F. McQuesten; New Jersey, S. Lilley; New York, H. W. Dean; North Carolina, R. J. Hicks; Ohio, A. Dunlap; Pennsylvania, W. J. Asdale; Rhode Island, L. Morton; Tennessee, W. T. Briggs; Texas, D. R. Wallace; Virginia, T. D. Cunningham; West Virginia, G. Baird; Wisconsin, E. P. Russell; United States Army, B. A. Clement; United States Navy, E. Eversfield.

Dr. J. B. Johnson, Chairman of the Committee of Arrangements, recommended, among others, the following gentlemen for membership: Drs. Wm. Vansant, J. W. Hollowbush, John Shore, B. Y. Edwards, St. Louis county; Daniel Lichty, Illinois; G. L. Pope, Arcadia, Illinois; Dr. Grimes, Kentucky; Edward Blakesly, Iowa.

Dr. E. L. Howard, of Baltimore, Chairman of the Special Committee, to whom had been referred the subject of a better organization of the Sections at the previous annual meeting, made a report, recommending the reorganization of Sections, as follows:

1st. Practice of Medicine, Materia Medica, and Physiology.

2d. Obstetrics, and Diseases of Women and Children.

3d. Surgery and Anatomy.

4th. Medical Jurisprudence, Chemistry, and Psychology.

5th. State Medicine and Public Hygiene.

The Committee also recommended that the Chairman of each Section be requested annually to deliver an address to the General Sessions, on the topics properly embraced in the Section over which he presides; and that the Standing Committees on Medical Education, Medical Literature and Climatology and Epidemics be abolished.

The same Committee further recommended that the Committee on Ethics be increased to nine members, and that the same be nominated by the Nominating Committee.

On motion of Dr. N. S. Davis, so much of the report of the Committee as related to the reorganization of the Sections was adopted.

On motion of Dr. J. R. Brenson, that part of the report in relation to Addresses by the Chairman of the Sections, and the omission of certain Standing Committees, was also adopted.

For that recommendation of the Committee relating to the appointment of a Committee on Ethics, Dr. N. S. Davis, moved the following by-law as a substitute, which was adopted:

XI—JUDICIAL COUNCIL.

A Council, consisting of twenty-one members, shall be appointed by the Nominating Committee, whose duty it shall be to take cognizance of and decide all questions of an ethical or judicial character that may arise in connection with the Association. Of the twenty-one members of the Council first appointed, the seven first named in the list shall hold office one year, and the second seven named shall hold office two years. With these exceptions, the term of office of members of the Council shall be three years, seven being appointed by the Nominating Committee annually.

The said Council shall organize by choosing a President and Secretary, and shall keep a permanent record of its proceedings. The decisions of said Council on all matters referred to it by the Association shall be final, and shall be reported to the Association at the earliest practical moment.

All questions of a personal character, including complaints and protests, and all questions on credentials, shall be referred at once—after the report of the Committee of Arrangements, or other presentation—to the Judicial Council, and without discussion.

Dr. Woodward, of the U. S. A., presented a communication concerning the injustice of the present regulations in regard to the rank of Surgeons and Assistant Surgeons in the army, and asked the aid of the Association in procuring such action of Congress as would remedy the evil. On motion of Dr. J. M. Keller, of Louisville, the communication was ordered on file, and a committee of five, appointed to memorialize Congress in behalf of the objects set forth therin.

The Secretary read the following appointments made by the President to represent the American Medical Association to the British Medical Association: Drs. F. G. Smith, C. Wister, J. S. Cohen, of Philadelphia; Dr. E. Warren, of Baltimore; Dr. C. L. Ives, of New Haven; Dr. Edward Montgomery, of St. Louis, and Drs. F. Barker, E. Seguin and J. C. Hutchison, of New York.

The Secretary stated that commissions for these gentlemen would be made out at once.

Dr. Johnson, of St. Louis, Chairman of the Nominating Committee, made the following partial report:

Your committee suggest the following gentlemen for the various offices named:

*President*, Dr. J. M. Toner, District of Columbia; *First Vice President*, W. Y. Gadbury, of Mississippi; *Second Vice President*, J. M. Keller, of Kentucky; *Third Vice President*, W. C. Husted, of Missouri; *Fourth Vice President*, L. D. Warner, of Massachusetts; *Treasurer*, Dr. Casper Wistar, of Philadelphia; *Librarian*, Wm. Lee; *Committee on Libraries*, Johnson Elliott; *Secretary*, Theo. A. McGraw; *Committee on Arrangements*, Dr. Brodie, Chairman; James A. Brown, Morse Stewart, J. F. Noyes, E. W. Jenks, Henry F. Lyster, D. O. Farrand, Eugene Smith, all of Detroit; *Committee on Prize Essays*, Drs. J. K. Johnson, A. Sager, H. Hitchcock, of Detroit; E. Andrews, Illinois; E. S. Gallard, Kentucky; *Committee on Publications*, Drs. F. G. Smith, W. B. Atkinson, D. Morrey Chester, of Pennsylvania; Wm. Lee, District of Columbia; Casper Westar, Pennsylvania; H. F. Askew, Delaware; Alfred Stilli, Pennsylvania.

Detroit was named for the next annual meeting of the Association.

The report was adopted.

Reports from the Standing Committees on Medical Education and Medical Literature were read and referred to the Committee of Publication.

Dr. John S. Moore, Chairman of the Committee on Prize Essays, reported that only one essay had been received by the Committee, and though containing much that was of interest and value, yet it was not possessed of that high order of merit which would justify the award of a premium.

The annual reports of the Committee of Publication and of the Treasurer were read and referred to the Committee of Publication.

Dr. J. M. Toner, of Washington, presented interesting statistical tables in reference to

Medical Societies and Public Hospitals in the United States, which were received and referred to the Committee of Publication.

Dr. F. W. Peck, of Davenport, Iowa, offerred the following preamble and resolution:

In view of the fact that the reports of Surgeon General of the United States Army, as exhibited in volumes one and two of the first part of the Medical and Surgical History of the War of the Rebellion, have received a too limited circulation by reason of an insufficient issue of the same by Congress, therefore

*Resolved*, That the President and Secretary of this Association be directed to petition Congress at the next session in behalf of the profession, asking that the edition recently issued be reproduced in sufficient number to permit of general distribution to the members of the profession throughout the country.

*Resolved*, That the thanks of this Association are due and are hereby tendered Congress for aiding thus far in developing and presenting to the profession reports of the Surgeon-General, as herein specified.

*Resolved*, That the thanks of this Association are hereby tendered the officers of the United States army who have, by sacrifice and labor, been instrumental in placing before the profession the valuable information contained in volumes one and two of the first part of the Medical and Surgical History of the War of the Rebellion.

Carried unanimously.

On motion the Association adjourned.

---

THIRD DAY'S SESSION.

The members were called to order at 9 o'clock, A. M., the President, Dr. Thomas M. Logan, in the chair.

The Permanent Secretary presented the Report of the majority of the Committee on the Nomenclature of Diseases, with resolutions appended asking for its adoption.

Dr. J. J. Woodward, of the U. S. A., opposed the adoption of the nomenclature proposed by the Committee, and offered the following resolutions as a substitute for those just read by the Secretary, and they were adopted by the Association:

*Resolved*, That in the opinion of this Convention it is inexpedient to adopt the nomenclature and classification presented by the majority of the Committee on Nomenclature at the meeting in Philadelphia.

*Resolved*, That a committee of three be appointed by the President, whose duty it shall be to communicate the foregoing resolution to the proper Committee of the Royal College of Physicians of London, and to negotiate for the representation of the American Medical Association in the first decennial revision of their nomenclature.

The Committee on Nominations, to whom was referred the subject of permanent membership, reported through their Chairman, Dr. J. B. Johnson, the following names for admission to the association:

Edwin Stewart, Mendon, Michigan; G. L. Polk, Aeola, Illinois; T. W. Hollowbush. Warsaw, Illinois; John Shore, St. Louis; Wm, Van Zant, St. Louis; Daniel Lichty, Rochelle, Illinois; B. F. Edwards, Kirkwood, Missouri; L. A. Grimes, Lewis county, Missouri; C. W. Crary, St. Louis; L. T. Newman, St. Louis; George F. Center, Olney, Illinois; Drs. R. J. Mitchell, Lewis Wilcox, R. J. Allmond, W. C. Day, R. T. Cowan, and Edwin Blakesley, all of Illinois; John E. Faber, St. Louis; Dr. Randall, Illinois; Dr. Vinnedge, Lafayette, Indiana; Charles N. F. Ludwig, St. Louis.

On motion, the report was adopted.

Dr. Toner, of the District of Columbia, presented the following resolution in regard to an International Medical Congress:

*Resolved*, That in the opinion of this Association it would be an excellent opportunity, at the American Centennial, in 1876, for an International Medical Congress to consider, and, if practicable, to adopt, a uniform classification and nomenclature of diseases, to be used by the profession throughout the world.

The Committee on Ethics for the Session of 1872 reported the following items of unfinished business:

The Committee on Ethics, of 1872, report in relation to the case of Paluel de Maimen, against whose admission as a delegate in meeting at Philadelphia, last year, a protest was entered, as follows:

Dr. de Maimen held credentials as a regular delegate from the Westchester County Medical Society in New York, to the meeting of the Association in 1872. But at that meeting a protest against his admission was made, on the ground that he was on trial for unprofessional conduct in his local Society. The subject was referred to this Committee at so late a period of the meeting that it could not then be acted upon. From the evidence recently presented to our Committee, it appears that the trial of Dr. de Maimen is still unfinished in the Westchester County Medical Society; that said Society has formally withdrawn his credentials as delegate to this Association, and consequently no present action in the matter is required by this Association.

Adopted.

At a meeting of the Association in Philadelphia, May, 1872, objections were made to the admission of delegates from the Pathological Society of Berks county, Pennsylvania, on the ground of non-professional conduct on the part of many of the members of that Society. Time not permitting a full hearing of the case during that meeting of the Association, a report on it was postponed until the next meeting of the Association. In the meantime the accused parties were duly notified of the charges, and requested to make answer thereto. After a full investigation of the case, your Committee on Ethics, appointed in 1872, declare a sufficient number of the charges sustained to justify the recommendation that the said Society be not allowed a representation in this Association.

Adopted.

Dr. N. S. Davis, Chairman of the Special Committee appointed to consider the proposition to attach to the office of Permanent Secretary an annual salary of $1,000, reported adversely to the proposition, and the report was adopted.

Dr. Kellar, of Louisville, Kentucky, submitted the following resolution as a substitute for the former one:

*Resolved*, That the sum of $500 be appropriated as an honorarium for the services of the Permanent Secretary during the present year, provided such an amount remains in the treasury after paying necessary expenses,

Adopted.

Dr. Bell, of Brooklyn, New York, offered the following resolutions:

*Resolved*, That in the judgment of this Association the establishment of a National Sanitary Bureau, with relations to the General Government, similar to those of the Bureaus of Agriculture and Education, is highly desirable as a means of promoting sanitary science and protection of the public health.

*Resolved*, That this Association request of the United States Educational Bureau to so extend the scope of its inquiry as to include vital diseases and mortuary statistics in relation to local, meteorological and geological influences, and to disseminate the information so collected throughout the country.

The resolutions of Dr. Bell, were, on motion, referred to the Committee on State Medicine and Public Hygiene.

Dr. Pollock, of Pittsburg, read the following report:

The Committee appointed by the President, at the last meeting of the Association, to take into consideration the propriety of adopting the suggestions of the Committee on Medical Education, are fully impressed with the importance of the subject, and acknowledge the value of the suggestions offered. But we believe it wholly impracticable to carry into operation any law which does not meet the hearty approval of diverse interests connected with the teaching and practice of medicine; and while we have no doubt that this Association has grown to be a power in the profession, felt and recognized by all, yet, to make this power effective, its decisions should be calm and deliberate. Therefore, your Committee, after due deliberation, have concluded to recommend the adoption of the conclusion of the report of the Committee on Medical Education, which is as follows: That a Congress, composed of two members from each State and Territory, and one from each recognized medical college, all to be members of this Association, be appointed (or nominated by the Nominating Committee) at this present session. That said Committee, or Congress, shall meet three days previous at our next annual meeting, and that said Committee, or Congress, shall perfect a plan for some uniform system of medical teaching, which, when adopted by the Association; shall be the only recognized method of medical teaching in the United States.

This report led to a somewhat lengthy and animated discussion. First it was adopted, by a close vote; then a motion was made to reconsider the vote, which was done. Then a motion was made to lay it on the table; then that motion was withdrawn.

Dr. Kellar, who had seconded the motion to lay it on the table, contended that the gentleman who made the motion had no right to withdraw it without consent of the second. He did not consent, but insisted on the motion to lay the report on the table. The motion was put by the Chair and the report laid on the table by the Convention.

Dr. N. S. Davis, of Chicago, Chairman of the Committee appointed to devise and recommend some plan for securing a more complete report of the doings in the several Sections of the Association, respectfully reports the following resolutions, and recommends their adoption:

*Resolved*, 1. That the Committee of Arrangements for each Annual Meeting of the Association are instructed to secure the services of a sufficient number of stenographic reporters to have one in attendance on the Regular Session of each of the Sections in the afternoon as well as during the General Morning Sessions; that the Reports thus obtained be printed the same evening on slips or proof-sheets, in sufficient number to supply all the members of the Association in attendance early the following morning.

*Resolved*, 2. That the necessary expenses incurred in carrying into effect the foregoing resolution shall be paid from the Treasury of the Association in the same manner as other bills relating to the publication of the transactions.

*Resolved*, 3. That the Acting Secretary of each Section shall keep a file of so much of the Reports as relate to the business of the Section to which he belongs; and it shall be his duty to revise the same, carefully eliminating therefrom all that is unimportant, and arranging that which is found valuable and pertinent in proper form, he shall transmit the same to the Permanent Secretary, for the Committee of Publication, within twenty days after the adjournment of each Annual Session of the Association.

The report was adopted.

Dr. E. L. Howard offered the following resolution:

*Resolved*, That the Chairmen of the various Sections shall have free scope in the selection of subjects for addresses.

Adopted.

On motion of the Permanent Secretary, it was ordered that the next annual meeting convene in Detroit on the first Tuesday in June, 1874.

The Committee on Nominations, submitted the following Report, which was adopted :

CHAIRMEN AND SECRETARIES FOR 1874.

1. Practice of Medicine, Materia Medica and Physiology—Dr. N. S. Davis, of Chicago, Chairman, and Dr. Frothingham, of Ann Arbor, Michigan, Secretary.

2. Obstetrics, and Diseases of Women and Children—Dr. Theo. Parvin, of Indianapolis, Chairman, and Dr. Montrose A. Pallen, of St. Louis, Secretary.

3. Surgery and Anatomy—Dr. S. D. Gross, of Philadelphia, Chairman, and Dr. Alonzo Garcilon, of Maine, Secretary.

4. Medical Jurisprudence, Chemistry and Psychology — Dr. A. N. Talley, of South Carolina, Chairman, and Dr. E. L. Howard, of Maryland, Secretary.

State and Public Hygiene—Dr. A. N. Bell, of Brooklyn, Chairman, and Dr. A. B. Stuart, of Winona, Minnesota, Secretary; F. A. Ross, Alabama; D. A. Linthicum, Arkansas; T. M. Logan, California; R. G. Buckingham, Colorado; B. H. Catlin, Connecticut; L. P. Bush, Delaware; F. Howard, District of Columbia; N. F. Westmoreland, Georgia; H. A. Johnson, Illinois; J. F. Hibberd, Indiana; J. J. Angear, Iowa; D. W. Stormont, Kansas; L. Rodgers, Kentucky; S. Fitch, Maine; James Stewart, Maine; H. J. Bowditch, Massachusetts; R. C. Kedzie, Michigan; A. B. Stuart, Minnesota; J. W. M. Shattuck, Mississippi; John M. Hodgen, Missouri; J. H. Parsons, New Hampshire; E. M. Hunt, New Jersey; A. N. Bell, New York; U. A. B. Norcon, North Carolina; Wm. Clendenin, Ohio; A. M. Pollock, Pennsylvania; G. M. Lenore, Rock Island; R. A. Kenlock, South Carolina; W. K. Briggs, Tennessee; D. R. Wallace, Texas; J. L. Caball, Virginia; James Bromfield, West Virginia; H. B. Strong, Wisconsin; A. F. Woodward, Vermont; S. M. Bemis, Louisiana.

*Resolved,* That the Secretary of the Association be authorized to fill up all vacancies in the Committee from the States and Territories. Also, that Dr. A. N. Bell be President, and Dr. A. B. Stuart Secretary.

COMMITTEE ON NECROLOGY.

Chairman, Dr. A. Sager, Ann Arbor, Michigan, and Committee, remain as last year, excepting Dr. Alonzo Garcilon, of Maine, in place of Dr. D. McReese, deceased; Dr. W. McCabe, Kansas; J. W. H. Baker, Iowa; J. H. Wheeler, New Hampshire; —— Milligan, Minnesota ; W. M. Chambers, Illinois; G. Sutton, Indiana; A. J. Lands, Georgia; B. A. Vaugh, Mississippi ; George Mitchell, Ohio ; J. J. Woodward, U. S. A.; N. M. Dodson, Wisconsin; L. B. Yandell, Sen., Kentucky.

JUDICIAL COMMITTEE.

Three years—Drs. Brodie, Michigan; N. S. Davis, Illinois; E. L. Howard, Maryland; Wm. O'Baldwin, Alabama; Dr. Dean, New York; J. P. Logan, Georgia.

Two years—L. S. Joynes, Virginia; R. N. Todd, Indiana; Askew, Delaware; J. E. Morgan, District of Columbia; Daniel Lillie, New Jersey; S. M. Benham, Pennsylvania; Dr. Dunlap, Connecticut.

One year—Drs. Bartlett, Wisconsin; Powell, Illinois; Gale, Kentucky; Moses, Missouri; Hughes, Iowa; Bemnis, Louisiana; Cheever, Massachusetts.

The Committee appointed to memorialize Congress in relation to the rule of promotion and rank of Medical Officers of the United States Army, was announced as follows:

Drs. J. M. Keller, of Louisville; H. F. Askew, of Wilmington, Delaware; J. M. Toner, of Washington; Murphy, of Cincinnati, and N. S. Davis, of Chicago.

On motion, the Association adjourned.

---

FOURTH DAY'S SESSION.

The delgates and members were called to order at 9½ A. M., the President, Dr. Logan, in the chair.

A resolution was passed that Dr. W. F. Peck, of Davenport, Iowa, be appointed a committee to report upon the treatment of injuries from railroad accidents.

The majority of the Committee on Ethics, through Dr. Davis, reported as follows on the protest of the St. Louis Medical Society against the registration of Dr. Adam Hammer, of St. Louis, as a permanent member of the American Medical Association:

That after due investigation and the hearing of both parties it appears that about one year since Dr. Adam Hammer was regularly arraigned and tried by the St. Louis Medical Society, of which he was a member, on the charge of violating the code of ethics. After an apparently full investigation he was declared guilty of the charges, and at a subsequent meeting he was suspended from membership for five years, or until reinstated by a vote of the Society. From this decision of the Society the suspended member appealed to a civil court for legal process to compel the Society to reinstate him. The Judge of the court decided that the charter of the Society gave it no authority to take cognizance of any personal assaults or controversies between its members, and issued, a peremptory *mandamus* requiring the Society to rescind its vote suspending Dr. Hammer, and to restore him to his rights of membership. Under this compulsory legal restoration to membership in the St. Louis Medical Society, Dr. Hammer claims the right to be registered as a permanent member at this meeting of the Association. After due consideration, it appears clear to a majority of the members of your Committee that the *mandamus* of the civil court has no other effect than simply to prevent the St. Louis Medical Society from enforcing any penalty against one of its convicted members, and does not in any degree affect the fact that he still stands convicted of having violated an important provision in the code of ethics. The constitution of this Association, in the clause defining who shall be *permanent members*, states "that permanent members shall consist of all those who have served in the capacity of delegates, and of such other members as may receive the appointment by unanimous vote, and shall continue such so long as they remain in good standing in the body from which they were sent as delegates."

Inasmuch as Dr. Hammer still stands convicted of a violation of the code of ethics by the St. Louis Medical Society, and said Society is restrained from inflicting the penalty it has adjudged proper for the offense, solely by the interference of a civil court, it cannot be claimed that he now remains in *good standing* in that Society.

Therefore your Committee is of the opinion that Dr. Hammer ought not to be received as a permanent member of this Association at its present meeting.

Signed by Drs. N. S. Davis, H. F. Askew, and D. W. Yandell.

Dr. E. Loy Howard made the following minority report:

The undersigned reluctantly dissents from the Report of the majority of the Committee on Ethics in the case of Dr. Hammer on the following grounds:

The only question before the Committee is, shall Dr. Hammer be permitted to register as a permanent member?

The constitution of this Association says, "The permanent members shall consist of all those who have served in the capacity of delegates, and of such other members as may receive the appointment by unanimous vote, and shall continue such so long as they remain in good standing in the body from which they were sent as delegates, and comply with the requirements of the by-laws of the Association."

It appears that Dr. Hammer is still a member of the St. Louis Medical Society, with all the rights and privileges of any other member of that body. The undersigned holds that the Committee have no right to examine the case beyond this record.

*Resolved*, That the name of Dr. Hammer be ordered entered as a permanent member of the Association.

Dr. Hammer maintained that, being accused, he was entitled to a hearing; but he was cut off by the "previous question," when the majority report was adopted by a large vote.

Dr. Frederick Horner, Jr., of Virginia, a surgeon in the United States Navy, presented the following resolution, with a strong appeal in favor of temperance :

*Resolved*, That in view of the alarming prevalence and ill effects of intemperance, with which none are so familiar as members of the medical profession, and which have called forth from eminent English physicians the voice of warning to the people of Great Britain, concerning the use of alcoholic beverages, that we, the undersigned members of the medical profession of the United States, unite in the declaration that we believe alcohol should be classed with other powerful drugs; that when prescribed medicinally it should be done with conscientious caution and a sense of great responsibility.

*Resolved*, We are of opinion that the use of alcoholic liquors as a beverage is productive of a large amount of physical and mental diseases ; that it entails diseased appetites and enfeebled constitutions upon offspring ; and that it is the cause of a large percentage of the crime and pauperism of our cities and country.

*Resolved*, That we would welcome any change in public sentiment that would confine the use of intoxicating liquors to the uses of science, art, and medicine.

Without debate, these resolutions were referred to the Committee on Public Hygiene and State Medicine.

The Nominating Committee made the following report finally :

The Committee recommend that the nomination of the Committee from the thirteen original States be deferred to the next annual meeting of the Association.

The Committee request the Convention to reconsider its action in the appointment of Dr. Myers, of Indiana, as a member of the Committee on Hygiene and State Medicine; and that the name of Dr. James F. Hibbard, of Indiana, be substituted.

The Committee also nominate Dr. W. O. Smith of Newport, Ky., as Chairman of a Special Committee on Perpetual Fever.

The report was adopted.

After the usual reports from the several Sections, the adoption of the customary complimentary resolutions, and some remarks from the President, the Association adjourned *sine die*.

